# Radical treatment of severe open fractures of extremities by orthoplastic surgery: a 10-year retrospective study

**DOI:** 10.1186/s13018-021-02479-2

**Published:** 2021-05-27

**Authors:** Zhao Yang, Chao Xu, Yong-Gang Zhu, Jun Li, Zi-Xiang Wu, Ji-Wei Zou, Bao-Bao Xue, Dan-Min Miao, Lei Shang, Guang-Yue Zhao

**Affiliations:** 1 Department of Military Medical Psychology, Air Force Military Medical University, No. 169 Changle Xi Road, Xi’an, Shaanxi Province 710032 People’s Republic of China; 2 Department of Orthopeadic Surgery, Xijing Hospital, Air Force Military Medical University, No. 127 Changle Xi Road, Xi’an, Shaanxi Province 710032 People’s Republic of China; 3 Department of Health Statistics, Faculty of Preventive Medicine, Air Force Military Medical University, No. 169 Changle Xi Road, Xi’an, Shaanxi Province 710032 People’s Republic of China

**Keywords:** Soft tissue reconstruction, Severe open fractures, Orthoplastic, Masqualet

## Abstract

**Objective:**

This study aimed to retrospectively analyze clinical data of a series of patients with severe open fractures of extremities (Gustilo IIIb or IIIc), who achieved a satisfactory outcome through radical orthoplastic surgery, so as to provide a reference for determining the treatment of severe open fractures of extremities.

**Methods:**

The clinical data of 41 consecutive patients with severe open fracture (Gustilo IIIb or IIIc) of the limb, who underwent successful surgical debridement, fixation, and soft tissue reconstruction in one stage between January 2008 and January 2019, were retrospectively reviewed. Postoperative indicators, including infection rate and union time, were acquired by a regular follow-up and analyzed.

**Results:**

The mean (±SD) age of the patients was 38 ± 16 years. A total of 90 open fractures and severe soft tissue damages were analyzed. The soft tissue cover was achieved within 72 h. The overall rate of infection was 14.6% (6/41). Sex and the Mangled Extremity Severity Score were associated with infection. The median union time of 40 patients (one amputation) was 32 weeks.

**Conclusion:**

The overall rate of infection exhibited a lower tendency in this study compared with previous studies on high-grade open fractures following a two-stage orthopedic approach. The consequence of infection rate and union time was similar to that in previous studies. These results indicated that the single-stage radical orthoplastic treatment was an effective and reliable option for reconstructing severe open fractures.

**Supplementary Information:**

The online version contains supplementary material available at 10.1186/s13018-021-02479-2.

## Introduction

It has been 20 years since Gopal and Smith et al. [1] published their remarkable achievements in dealing with severe open fractures of the tibia by radical orthoplastic approach. However, similar studies have rarely been reported in recent years. This is mainly because severe open fractures (referred to as Gustilo IIIb or IIIc injuries), which often lead to large soft tissue defects and high risk of infection, are still a challenge for reconstructive surgeons [2, 3]. Although various methods and standards have been used for managing open fractures in the lower limb [4–6], the salvage treatment is still debatable in severe cases [7].

The present popular concept of severe open fracture management aims to achieve soft tissue coverage in an early stage. It is based on the collaboration of orthopedic and plastic (microvascular) surgeons in an “orthoplastic” central unit [8]. Compared with the traditional orthopedic approach in which the primary stabilization of the fracture and delayed wound closure are completed in two stages, the combined “orthoplastic” treatment has advantages such as fewer flap failures, lower infection rate, decreased bone-healing time, and short hospital stay [1, 4].

Despite remarkable superiority, orthoplastic treatment is not used worldwide yet. Especially in Mainland China, the traditional orthopedic approach is generally accepted to deal with severe limb trauma. However, several surgeons in the orthopedic department of Mainland China can handle both fixation and microsurgery. This is quite different from the “orthoplastic center” mode in the UK [6].

Besides, the orthoplastic approach proposed by Gopal et al. [1] is relatively radical compared with the “orthoplastic” treatment recommended by the British Orthopaedic Association and the British Association of Plastic Reconstructive and Aesthetic Surgery [6]. The major difference is whether the soft tissue cover is achieved in a single primary procedure. The current popular opinion holds that immediate soft tissue cover is not safe [3, 9]. In contrast, staged surgery for early coverage within 72 h is relatively safe and stable [10]. Nevertheless, Gopal et al. [1] showed excellent union and low rates of infection in aggressive management, proving its effectiveness and operability.

Based on the aforementioned findings, our department made a series of attempts in dealing with severe open fractures of limbs using the “radical orthoplastic” treatment [1] since September 2008. This study aimed to evaluate the infection rates and union time retrospectively in patients who had Gustilo-Anderson grades IIIB and IIIC open fractures of limb and accepted a single-stage orthoplastic treatment. It was hypothesized that the “radical orthoplastic” approach would be an effective treatment.

## Methods

### Patients

The data of 41 patients suffering from severe limb injury and undergoing successful surgery, including debridement, fixation, and soft tissue reconstruction, in one stage in the Xijing Hospital between January 2008 and January 2019, were retrospectively reviewed using the medical and follow-up records. The injury was confined to upper and lower limbs, including 90 open fractures and severe soft tissue damage (mainly types IIIB and IIIC, according to Gustilo criteria) [3]. These cases were followed up consistently. Unfortunately, one patient came to an end of amputation due to personal economic reasons.

### Treatment protocol

The treatment protocol was as follows: Patients underwent temporary stabilization with cast and life-supporting treatments as appropriate on arrival at the emergency department of the Xijing Hospital. Further procedures in the orthoplastic approach were started as quickly as possible when the patient was transferred to our department of orthopedic surgery. Immediate radical wound debridement and transitional fixation were performed for those with grade IIIB injury. Profuse lavage was used, and the debridement area exceeded the injury zone. Skeletal stabilization was achieved with a transitional plate (usually a short and thin tubular plate is used to reduce the infection risk) or external fixation or screw or a combination of these depending on the anatomy of the fracture. More critically, the soft tissue defect was immediately reconstructed using a vascularized muscle flap with a split skin graft or a local transfer flap according to the anatomy of the injury rather than by temporary negative pressure dressing. For the grade IIIC injury, the vascular reconstruction was accomplished first, and the protocol followed was the same. Masqualet or bone-shortening methods were used for solving bone defect problems in some patients. A delayed operation of bone graft and plate replacement (with a rigid reconstruction plate) was done 7–29 weeks (at a mean time of 13 weeks) later when all infection indicators were normal.

Postoperative rehabilitation included intravenous antibiotics (cefoperazone sodium and metronidazole), which were administered for the first 5 days. Furthermore, antibiotic treatments were adjusted according to the indications from cultures from the areas of the superficial skin graft. All patients were advised for joint movements on bed. A routine postoperative anticoagulant, anticonvulsant, anti-infective therapy was performed to ensure the survival of the flap. Partial weight-bearing was permitted until 12 weeks postoperatively after early bony stability was obtained. External fixation was removed 3 months after the surgery as appropriate.

Besides, the Masqualet bone cement technique was introduced since August 2017, which provided not only defect fillings but also an antibacterial effect on the fracture site.

### Follow-up

**Fig. 1:**
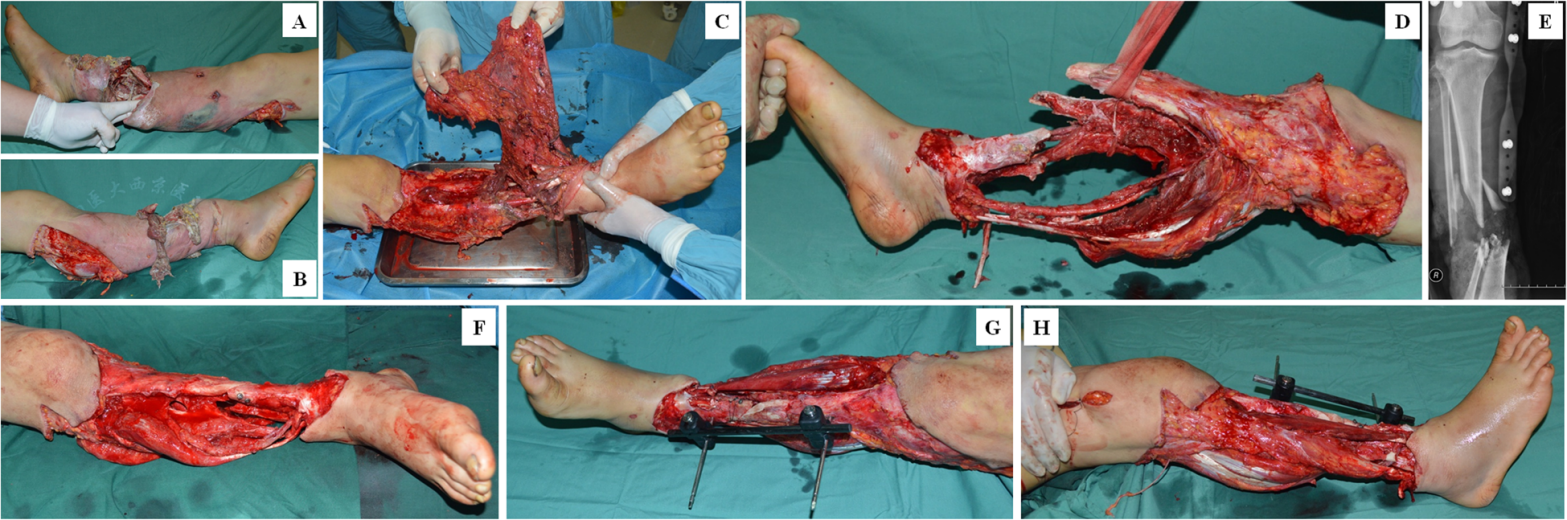
Case 1-1. Appearance of right leg due to a blunt injury. (**A**-**C**). Appearance of right leg after debridement (**D**). Radiographic examination of right tibial and fibular fractures (**E**). Appearance of right leg after shortening and external fixation (**F**-**H**)

**Fig. 2:**
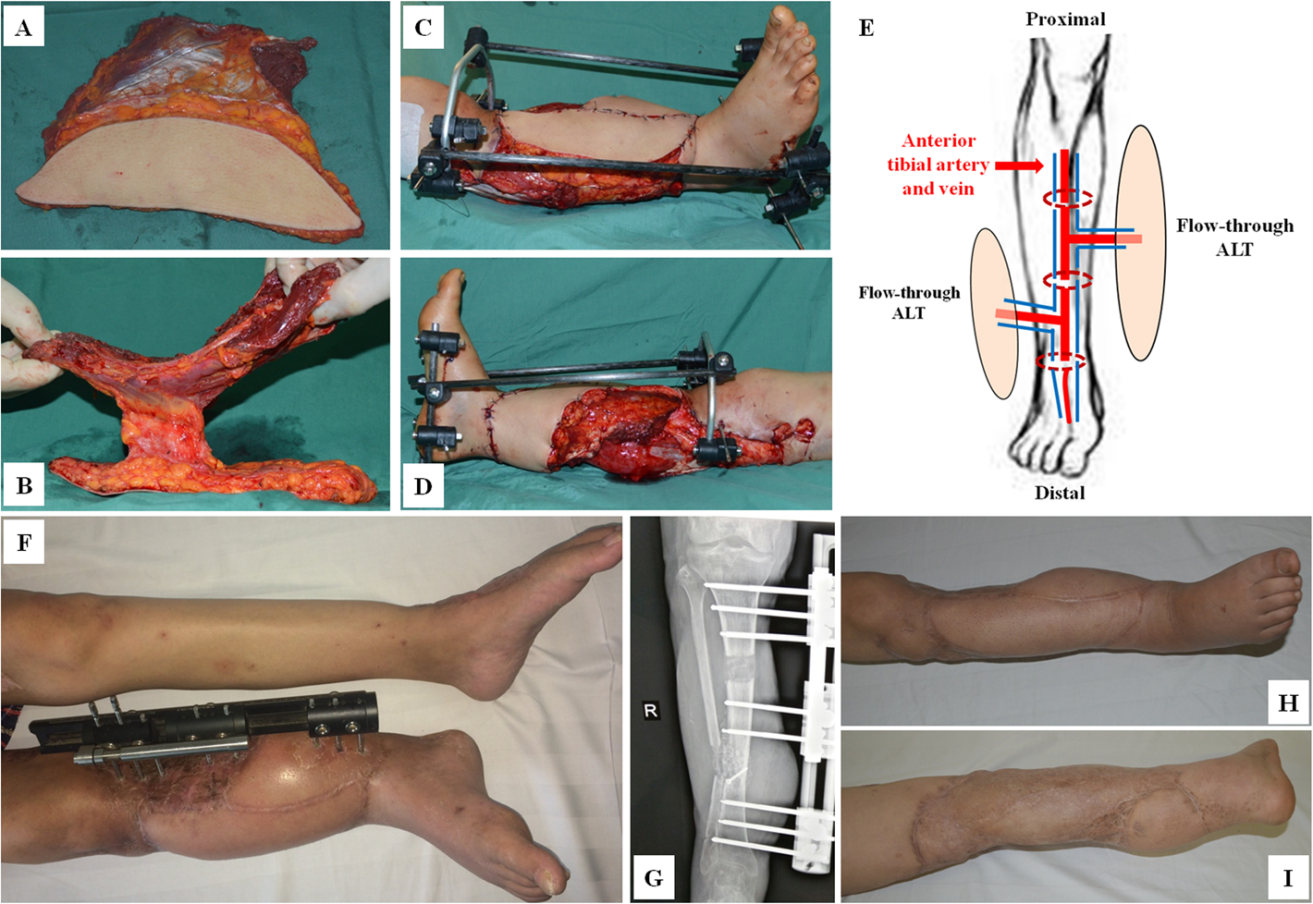
Case1-2. The design of double flow-through ALT flaps for right leg reconstruction. Appearance of left ALT flap (**A**) and right ALT flap (**B**). Defects covered by double ALT flaps (**C** and **D**). Diagram of double flow-through ALT flap (**E**). The appearance of survived composite flap in the right leg at 6 months postoperation (**F**). X-ray examinations of tibial osteotomy lengthening (**G**). Appearance of the survived composite flap at 9 months follow-up (**H**-**I**)

**Fig. 3:**
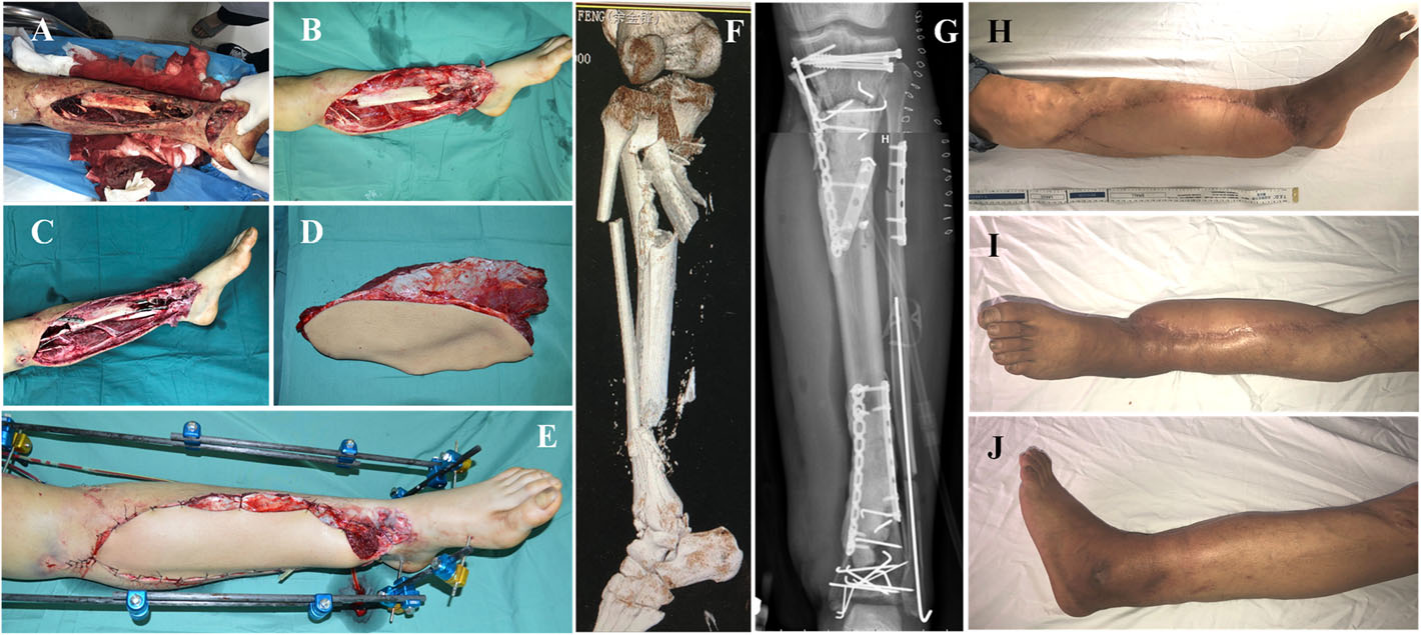
Case 2. Appearance of left leg due to a bruise injury. (**A**). Appearance of left leg after debridement (**B**). Appearance of left leg after fixation (**C**). Appearance of right ALT flap (**D**). Intraoperative appearance after flap transfer (**E**). Radiographic examination of left tibial and fibular fractures (**F**). Appearance of left leg after internal fixation and bone cement filling (**G**). Appearance of the survived composite flap at 9 months follow-up (**H**-**J**)

Patients discharged from the hospital were appointed in the orthopedic clinic for subsequent follow-ups until the union of the fracture. All participants were interviewed and checked by the surgeon responsible for the whole medical process. The examinations included the following: X-ray for bone-healing observation, status of soft tissue recovery, and any abnormal appearance of the reconstructed limb. Due to clinical suspicion, positive skin and bone tissue cultures and routine blood tests were performed, and high-sensitivity C-reactive protein level and erythrocyte sedimentation rate were determined. Hybrid positron emission tomography-computed tomography (PET/CT) bone scanning was used in the case of suspected osteomyelitis. The follow-up time ranged from 6 to 36 months postoperatively according to the rehabilitation of the patient (Figs. 1, 2, and 3).

### Data collection

Injury details included Mangled Extremity Severity Score (MESS), open fracture classification, AO fracture classification, device of stabilization, flap type, initial antibiotic timing, and timing of flap. The results were obtained from the surgeon who was responsible for the entire operation.

### Statistical analysis

Quantitative variables were presented as means ± standard deviation. Medians with interquartile range (IQR, presented as the upper and lower quartiles) were used when the data were skewed, and qualitative variables were expressed as frequency and percentages [*n* (%)]. Fisher’s exact probability test was used for categorical variables (Table 4). Moreover, *t* tests were used for continuous variables with normal distribution in the two groups; Mann–Whitney *U* test was used for continuous variables when the data were skewed (Table 4). All statistical analyses were performed using Statistical Package for the Social Sciences (SPSS) 16.0 (SPSS, IL, USA). A *P* value less than 0.05 was considered statistically significant.

## Results

A total of 41 patients with 90 severe open fractures [89 IIIb (98.9%) and 1 IIIc (1.1%)] were examined. The mean (± SD) age was 38 ± 16 years, 75.6% (31 cases) were men, and the injuries were due to traffic accidents (28 cases, 68.3%), blunt trauma (9 cases, 22%), drifting-down injury (3 cases, 7.3%), and twist trauma (1 case, 2.4%). All the patients were followed up to a minimum of 2 year since the end of their clinical course. One patient, unfortunately, selected amputation 4 weeks after the primary surgery because of economic reasons. The description of the demographics and injury details are shown in Table 1. Injury details, including MESS, open fracture classification, AO fracture classification, device of stabilization, flap type, initial antibiotic timing, and the timing of flap, were analyzed.

**Table 1:** Demographics and clinical details of the patients

Age (years)	Gender^△^	MESS score	Gustilo classification	AO classification	Fixation^*^	Flap^#^	Initial antibiotic timing (h)	Flap timing (h)	Secondary procedures^&^	Union (weeks)
20	F	6	IIIB	42B3(b), 4F2A(b)	EF	ALT	12	18		45
54	M	10	IIIB	43C3.3, 4F2B(b),44B3.3	ORIF	ALT	8	14	Plate replacement 6 weeks	52
42	F	7	IIIB	13C3.3, 2U1B1	ORIF	ALT	24	35	Skin graft 6 weeks	43
34	M	7	IIIB	43A3.2, 4F3A	EF	ALT	9	18		15
31	M	8	IIIB	42C3(j), 4F2B(b)	ORIF + Screw	ALT	13	56	EF 6 weeks	84
44	M	6	IIIB	2R3A3.1, 2U3A2.3, 77.2.1A, 77.3.1A	ORIF	ALT	4	18		29
9	F	6	IIIB	43A1.2, 4F2A(c), 87.1.3C	EF + Screw	ALT	22	37		16
47	M	10	IIIB	42B2(b), 4F2A(b)	EF + Screw	GAS	6	16		35
51	F	11	IIIB	42B3(a), 4F1B(n)	EF	GAS	20	30	Skin graft 6 weeks	35
62	F	10	IIIB	42B3(a), 4F2A(a)	EF	GAS	12	18		39
59	M	11	IIIB	42C3(i), 4F2A(b)	EF	SOL	14	20	Bone graft + plate replacement + flap 19 weeks	40
32	M	11	IIIB	42C2(j), 4F3A	EF + Screw	ALT	11	24	Flap 12 days	70
45	M	6	IIIB	42B2(a), 4F2A(a)	EF + Screw	GAS	6	16		36
41	M	11	IIIC	2R2C3(j), 2U2C3(j)	EF	ALT	4	16	Bone shortening + plate 26 weeks	52
24	M	8	IIIB	42B2(c), 4F2A(c)	EF	ALT	12	50	Plate 9 days	38
41	M	7	IIIB	42C3(j), 4F2B(a)	EF	SOL	14	21		30
42	M	7	IIIB	42C3(j), 4F2A(b)	EF	SOL	3	12	Plate 8 weeks	32
8	F	6	IIIB	42C2(j), 4F2B(b)	EF + Screw	ALT	24	39	Plate 6 weeks	18
39	F	10	IIIB	42C3(j), 4F2B(b)	EF + Screw + Bone shortening	F-T ALT + ALT	26	29	Bone lengthening 35 weeks	62
17	F	9	IIIB	2U2A2(b)	EF + ORIF	ALT	8	20		12
32	F	7	IIIB	43B1.1	EF	ALT	13	21		12
22	M	9	IIIB	42A2(a)	EF	GAS	13	20	Flap 4 weeks	12
37	M	7	IIIB	42A3(b), 87.1.1B	EF + Screw	SOL	18	27		32
44	M	9	IIIB	42B2(c), 4F3B	EF + ORIF + Cement	F-T ALT	18	22	Bone graft + plate replacement 8 weeks	22
54	M	11	IIIB	42C3(k), 4F3B, 81.1.B2, 82A2	EF + ORIF + Screw	ALT	20	33	Bone graft 7 weeks	55
50	M	10	IIIB	42C2(i), 42A2(b), 4F1A(n), 4F2A(b)	EF + ORIF+ Screw	GAS	20	31	Skin graft 4 weeks	36
65	M	8	IIIB	42B2(c), 4F2A(b)	EF + ORIF + Screw	SOL	11	19	UTN + bone graft 21 weeks	47
9	F	6	IIIB	42B2(b)	EF + ORIF	SOL	12	17	Bone graft + plate replacement 29 weeks	42
43	M	9	IIIB	42C3(i), 4F2A(a), 87.1.1B, 87.3.2A	EF + ORIF + Screw	SOL	30	42	Amputation	-
46	M	7	IIIB	42C3(j), 4F2B(b)	EF + ORIF + Screw	SOL	18	26	Bone graft + plate replacement 11 weeks	30
33	M	7	IIIB	42C2(j), 4F2A(b)	EF + Screw	ALT	11	21	Bone graft + plate replacement 7 weeks	23
27	M	6	IIIB	41B3.1, 42C3(j), 4F2A(a)	EF + ORIF + Cement	ALT	12	28	Bone graft + plate replacement 17 weeks	30
24	M	4	IIIB	42C3(j), 4F2A(b)	EF + ORIF + Cement	GAS	23	31	Bone graft + plate replacement 17 weeks	29
30	M	8	IIIB	42C3(j), 4F2B(b)	EF + ORIF + Cement+ Bone shortening	GAS	14	27	Bone graft + plate replacement 10 weeks	18
42	M	7	IIIB	42C2(j), 4F2B(b)	EF + ORIF + Cement	SOL	15	25	Bone graft + plate replacement 7 weeks	21
77	M	9	IIIB	32B3(c), 42B3(b)	ORIF + Screw + Cement	GAS	6	18	Skin graft 2 weeks	50
27	M	6	IIIB	42C3(j), 4F2A(b)	EF + ORIF + Cement	SOL	9	17	Bone graft + plate replacement 7 weeks	19
20	M	6	IIIB	41C3.3, 4F2A(a)	EF + ORIF + Screw + Cement	GAS	11	19		23
65	M	8	IIIB	42C3(j), 4F2A(b)	EF + ORIF + Cement	SOL	25	38	Bone graft + plate replacement 8 weeks	32
37	M	9	IIIB	43A2.1, 44B3.1	EF + ORIF + Screw	ALT	7	41	Skin graft 3 weeks	33
39	M	7	IIIB	41C3.1, 42C3(j), 4F2A(a)	EF + ORIF + Screw + Cement	SOL	20	33	Bone graft + plate replacement 18 weeks	32

^△*F* female, *M* male^

^*EF external fixation, *ORIF* plating, *Cement* bone cement^

^#*ALT* anterolateral thigh flap, *GAS* gastrocnemius, *SOL* soleus^

^&*U*^*TN* unreamed tibial nial

### Fixation

Table 2 exhibits the kind of fixation device chosen for different patients and associated results. Nine options were chosen for different fractures and 7 of them were applied in a multiple fixation devices combinational way rather than used solely.

**Table 2:** Details of the results according to the fixation device used

Fixation device	Number	Amputation	Union time (median, IQR, weeks)	Acute-flap infection	Chronic-fracture site infection
EF	11		35 (15 to 40)		1
ORIF	3		43 (29 to 52)		1
EF + ORIF	2		27 (12, 42)		
EF + Screw	8		33.5 (19.25 to 55.5)		1
ORIF + Screw	1		84	1	
EF + ORIF + Screw	6	1	36 (31.5 to 51)		1
ORIF + Screw + Cement	1		50		1
EF + ORIF + Cement	7		22 (19 to 30)		
EF + ORIF + Screw + Cement	2		27.5 (23, 32)		

### Soft tissue reconstruction

The soft tissue cover was accomplished by two kinds of local transfer flaps (10 gastrocnemius and 12 soleus) and free anterolateral thigh flap (19 cases) according to the characteristic of injury (Tables 1 and 5). All flaps survived in all patients uneventfully and showed better appearance, color, and texture as well as satisfactory sensation. More significantly, the soft tissue cover was achieved within 72 h in all 41 patients, and the median (IQR) time was 22 (18–32) h. Furthermore, 22 cases were covered immediately (≤ 24 h), while the remaining as early as possible (24–60 h). The associated results between immediate and early coverage are shown in Table 3.

**Table 3:** Details of the results related to the timing of soft-tissue cover

Timing of cover (h)	Number	Amputation	Union time (median, IQR, weeks)	Flap infection	Deep infection
Immediate (≤ 24)	22	0	33.5 (21.25 to 45.5)	0	4 (18%)
Early (24-60)	19	1	32 (27 to 39.25)	1 (5%)	1 (5%)

### Infection

The overall rate of infection was 14.6% (6/41), which included one acute flap infection and five chronic fracture site infection. The rate of infection was 28.6% (4/14) in the first 6 years (September 2008 to July 2014), and 7.4% (2/27) in the next 5 years (August 2014 to April 2019). These problems were resolved by antibiotics and repeated debridement. The distribution details of infection based on age, sex, MESS score, time of coverage, initial antibiotic timing, and characteristics of the injury are shown in Tables 4 and 5. Six infected fractures were found in men with a high MESS score (> 7). A comparison of the infected and noninfected fractures in Table 4 shows that males were associated with an increased rate of infection (*P* < 0.001). The same results were obtained for the increased MESS score (*P* = 0.021). However, no significant difference was found in age, time of coverage, and initial antibiotic timing between the infected and noninfected fractures.

**Table 4:** Factors associated with infection

Variable	Noninfected fractures (n = 35) (%)	Infected fractures (n = 6) (%)	***p*** value
Mean age (years) (range)	36 (8 to 65)	48 (31 to 77)	0.093
Male gender	25 (71.4)	6 (100)	**0.0001**
MESS > 7	15 (42.9)	6 (100)	**0.021**
Lower limb fracture	32 (91.4)	5 (83.3)	0.483
Median time to first coverage (h) (IQR)	22 (18 to 31)	21 (15.5 to 38.75)	0.592
Immediate coverage (≤ 24)	18 (51.4)	4 (66.7)	0.668
Initial antibiotic timing (h) (range)	14.7 (3 to 30)	10.3 (4 to 20)	0.139

Statistically significant analyses are highlighted in bold

**Table 5:** Characteristics of the soft tissue injury and coverage

Characteristic	Number	Infection (%)
Main injured zone		
Elbow joint	1	
Middle forearm	2	1 (50%)
Forearm to hand	1	
Proximal leg	3	
Middle leg	26	3 (11.5%)
Distal leg	2	
Distal leg to ankle/foot	6	2 (33.3%)
Soft tissue defect location		
Anterior	5	2 (40%)
Interior	4	1 (25%)
Anteromedial	15	1 (6.7%)
Anterolateral	4	2 (50%)
Posterior	1	
Posterolateral	1	
Extensive	11	
Flap		
ALT	19	5 (26.3%)
GAS	10	1 (10%)
SOL	12	

The rate of main injured zone infection at the site of the middle forearm, middle leg, and distal leg to ankle/foot was 50% (1/2), 11.5% (3/26), and 33.3% (2/6), respectively. For soft tissue defect location, the infection rate was 40% (2/5) anteriorly, 25% (1/4) interiorly, 6.7% (1/15) anteromedially, and 50% (2/4) anterolaterally. Besides, the rate of infection was 26.3% (5/19) in the free anterolateral thigh flap group and 10% (1/10) in the gastrocnemius flap group. Nevertheless, the number in each group was too small to identify a significant difference.

### Bone healing

The median union time of all 40 patients was 32 weeks (IQR, 22.25–42.75). The union time in the nine fixation device groups is shown in Table 3. The median union time in the group with immediate placement of the cover was 33.5 weeks (IQR, 21.25–45.5). However, the median union time in the group with early placement of the cover was 32 weeks (IQR, 27–39.25). The difference in union time between the two groups was not statistically significant (*P* = 0.87).

## Discussion

The UK orthoplastic concept emphasizes that early soft tissue coverage is usually accomplished within 72 h [11]. This is largely due to the joint care by orthopedic trauma surgeons and plastic surgeons with experience in limb reconstruction in their orthoplastic specialist center. Combined procedures and appropriate support services (including microbiologists, interventional radiologists, rehabilitation specialists, limb prosthetic services, and psychologists) are provided, which is their biggest advantage [6, 12–14].

However, patients undergoing this approach still face relatively less repeated debridements and delayed reconstruction of soft tissue. Gopal et al. [1] revealed a more aggressive orthoplastic management of the severe open fracture of the tibia. Similar attempts were reported in many previous studies [4, 15]. Despite the controversy regarding safety [9, 16, 17], such radical approaches based on immediate soft tissue cover are effective with excellent union and low rates of infection. Using this concept, soft tissue coverage is accomplished as a single primary procedure.

Currently, the traditional orthopedic approach with delayed wound closure is popular in China, making the salvage treatment of severe limb injury complicated and tougher for orthopedic surgeons. Moreover, no orthoplastic center has been built in China yet. However, numerous orthopedic surgeons are available in China who can deal with not only fractures but also soft tissue reconstruction, generating orthoplastic of Chinese characteristics-“orthoplastic surgeon.” Based on radical and thorough debridement by senior qualified surgeons, the aggressive orthoplastic management reported by Gopal et al. [1] has been used since 2008. Also, satisfactory outcome results have been observed.

The infection rate is the key monitoring target of postoperative complications. Posttraumatic infection is commonly observed in severe open fractures. In this study, the overall rate of infection was 14.6%, exhibiting a lower tendency compared with the results in other previous studies on high-grade open fractures following a two-stage orthopedic approach [13, 18]. More critically, the consequence was similar to the report of Gopal et al. [1] (with an overall rate of infection of 15.9% within 72 h) and an infection rate of 14.5% in a prospective multicenter cohort study of orthoplastic surgical collaboration [8]. However, two aspects are still worth introspection. First is that a more aggressive but thorough initial debridement was carried out by senior professors of our team. Second, the introduction of induced membrane technique using bone cement and antibiotics in the orthoplastic treatment since August 2017 deserved attention. This two-step procedure was highly superior in treating bone defects and nonunions [19–21].

In common with Khan [8] and Gopal [1], the median union time largely reflected excellent results of the proposed treatment. Nevertheless, hybrid fixation devices were used in most cases, which was different from the approach of Khan and Gopal. The reason was that greater stability reduced the risk of infection and nonunion of the fracture site. Moreover, a tubular plate was used in this study, which was short and thin to reduce the infection risk in initial orthoplastic treatment, and defined it as a transitional plate. Moreover, it was replaced with a rigid reconstruction plate 7–29 weeks later, when all infection indicators are normal. This procedure reduced the risk of infection and ensured the stability of the fracture.

Khan et al. [10] pointed out that the radical “fix-and-flap” approach proposed by Gopal et al. [1] might not be pragmatic or appropriate, and staged orthoplastic surgery was more optimized. We acknowledge that the fracture fixation and vascularized soft tissue cover in the first operation was a huge challenge for reconstruction surgeons. However, delayed coverage (even within 72 h) would have been even tougher with repeated debridement and was associated with a higher rate of deep infection [18]. On the other hand, gastrocnemius and soleus transfer flaps chosen for soft tissue reconstruction were easy to handle and had a high survival rate. Free anterolateral thigh flap also had significant benefits: vascular pedicle was longer, the main artery need not be sacrificed, a flow-through blood supply was provided (a successful case 1 is shown in supplementary materials, which has been previously published in Annals of Plastic Surgery by our team) [22, 23].

## Conclusion

In summary, the overall rate of infection exhibited a lower tendency in this study compared with previous studies on high-grade open fractures following a two-stage orthopedic approach. The consequence of infection rate and union time was similar to that in previous studies. This study indicated that the radical “orthoplastic” treatment was an effective and reliable option for the reconstruction of severe open fractures. The limitation of this study was that the focus was mainly on the infection rate and union time. Therefore, a prospective clinical trial was conducted to analyze the safety of the orthoplastic approach. The analysis included not only indicators used earlier but also a series of assessments for limb function and psychological states to make a comprehensive evaluation of individual recovery.

## Supplementary Information

**Additional file 1.** Supplementary data-Case
